# Effectiveness and Safety of Transcatheter Patent Foramen Ovale Closure for Migraine (EASTFORM) Trial

**DOI:** 10.1038/srep39081

**Published:** 2016-12-14

**Authors:** Ying-qi Xing, Yu-Zhu Guo, Yong-Sheng Gao, Zhen-Ni Guo, Peng-Peng Niu, Yi Yang

**Affiliations:** 1 Department of Neurology and Neuroscience Center, the First Hospital of Jilin University, Changchun, China; 2 Department of Cardiac Surgery, the First Hospital of Jilin University, Changchun, China; 3 Department of Neurology, Peking University Shenzhen Hospital, Shenzhen, China

## Abstract

We evaluated the safety and effectiveness of transcatheter patent foramen ovale (PFO) closure for the treatment of migraine in a Chinese population. This non-randomized clinical trial enrolled 258 consecutive substantial or severe migraineurs with a right-to-left shunt (RLS) (grade II–IV) and grouped subjects according to their election or refusal of PFO closure. Migraine was diagnosed according to the International Classification of Headache Disorders III-beta and evaluated using the Headache Impact Test-6 (HIT-6). In total, 241 participants (125 in the transcatheter closure group and 116 in the control group) were included in the study. In general, the PFO closure procedure was found to be safe. At 1 month after closure, 76.1% of patients returned for c-TCD evaluation; of these, 85.7% were downgraded to negative status or a grade-I shunt. Residual shunts and placebo effects were thought to resolve by 12 months post-procedure, when migraine impact was reported to decrease by 73.6%. Transcatheter PFO closure was demonstrated to be effective for the treatment of migraine by comparing HIT-6 scores between the transcatheter closure and control groups (p < 0.001). Our results suggest that transcatheter PFO closure is a safe and effective approach for the treatment of migraine in the Chinese population, especially in females with constant RLS. Clinical trial no. NCT02127294 (registered on April 29, 2014).

The prevalence of primary headache in the Chinese adult population is 23.8%, with migraine, tension-type headache, and chronic daily headache affecting 9.3%, 10.8%, and 1% of individuals, respectively^1^. The World Health Organization ranks migraine as the number seven cause of disability. Yet, a lack of information about the pathogenesis of migraine limits its effective treatment.

Patent foramen ovale (PFO) is the most common congenital defect of the atrial septum and affects about 25% of the general population^2^. During foetal development, the foramen ovale facilitates communication between the right and left atria, allowing oxygenated blood flow to avoid the nascent foetal lungs. In most individuals, the foramen ovale closes shortly after birth following a rise in left atrial pressure due to an increase in pulmonary blood flow after the initiation of breathing. However, in some individuals, the primum and secundum septa fail to fuse completely, resulting in PFO. In recent decades, independent reports have hypothesized an association between PFO and migraine, and especially migraine with aura^3^,^4^,^5^,^6^,^7^,^8^. The proposed mechanism of this type of headache is the arrival of agents into the cerebral circulation that are normally removed by the lungs, which subsequently triggers a migraine attack or lowers the threshold for migraine occurrence^9^. Observational studies have also reported reductions in the frequency of migraine attacks after percutaneous PFO closure; in studies, up to 90% of patients reported a reduction or cessation of migraine attacks after undergoing PFO closure for a non-migraine-related indication^10^,^11^,^12^,^13^. Although transcatheter closure of the PFO has been available for more than two decades, the use of this procedure has remained controversial due to a paucity of evidence to guide patient eligibility and device selection^14^. Recent contemporary studies have investigated PFO closure as treatment for patients with cryptogenic stroke, migraine, and orthodeoxia/platypnea, such that longitudinal data regarding the safety and efficacy of these devices is now available^5^,^8^,^15^,^16^,^17^,^18^,^19^,^20^. However, no study to date has investigated PFO closure for the treatment of migraine in the Chinese population.

The aim of the present study was to evaluate the long-term impact of PFO closure on migraine in a real-world setting of patients prospectively enrolled and included in a continuous registry at a single centre in China.

## Results

### Study population

**Figure 1:**
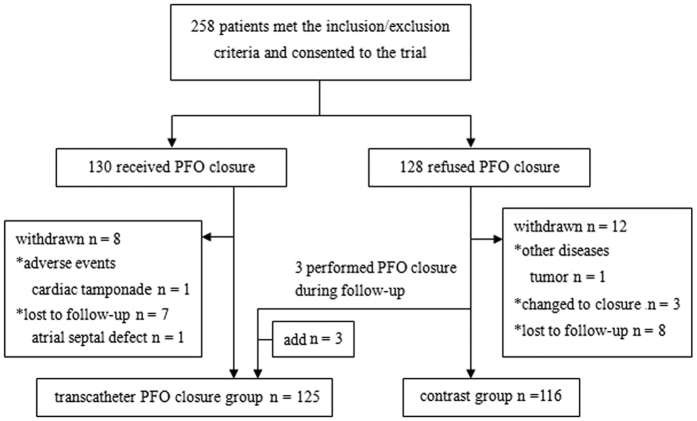
Typical patient disposition during the follow-up period.

In total, 241 participants (transcatheter closure group, n = 125; control group, n = 116) were included in the study. Participant demographic and clinical characteristics are summarized in Table 1. The groups were similarly matched in terms of age, gender, disease course, aura, type of right-to-left shunt (RLS), and baseline Headache Impact Test-6 (HIT-6) score. A typical patient disposition during the 1-year follow-up period is shown in Fig. 1.

### Procedural outcomes

A PFO occluder was successfully implanted in all 125 patients of the transcatheter closure group. Only one patient experienced cardiac tamponade, and no patients reported serious complications such as coronary artery air embolism, formation of a thrombus on the implant device, cardiac arrhythmia, thromboembolism related to the implant device, cardiac perforation, or infective endocarditis. The sizes of the Cardi-O-Fix PFO devices used were 18 mm (n = 1), 18–25 mm (n = 31), 25 mm (n = 66), 30 mm (n = 22), and 25–35 mm (n = 5). Three patients required transseptal puncture due to difficulty passing the guide wire through the PFO, while all other patients exhibited successful input through the PFO. Transthoracic echocardiography (TTE) after implantation showed that the position and shape of the occluder was optimal in all patients. Large residual shunts were observed by contrast-enhanced transcranial Doppler (c-TCD) in 18 patients in the first 3 days after the procedure.

### Follow-up safety evaluation of transcatheter PFO closure

Minor adverse events, including palpitations (18.0%), chest discomfort (4.9%), weakness (3.3%), and dyspnoea (1.6%) were reported within 1 month after the procedure in transcatheter closure patients. In cases where palpitations were reported, electrocardiography (ECG) was performed and did not reveal significant abnormalities in any of these cases. Adverse event-related symptoms resolved in most patients by the 6-month follow-up examination. Four patients reported severe visual aura (scintillating scotoma) with or without migraine after PFO closure, three of which had never experienced these symptoms before the procedure. Visual aura reduced or disappeared over time without any treatment.

### Follow-up efficacy evaluation of transcatheter PFO closure for migraine

#### Residual RLS detection during the follow-up period

**Figure 2:**
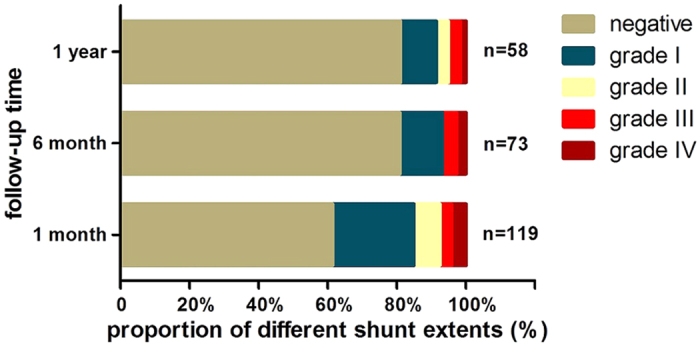
Changes in residual RLS status at different time points during the follow-up period in the transcatheter closure group.

During the 1-year follow-up period, a total of 121 patients in the transcatheter closure group returned for c-TCD. Of these, 119 returned for c-TCD one month after closure, and 46 (38.0%) had residual shunts at this time: five patients had a grade IV shunt, four had a grade III shunt, nine had a grade II shunt, and 28 had a grade I shunt. Three months after closure, 88 patients returned for c-TCD, and 25 (28.4%) had residual shunts: five patients had a grade III shunt, five had a grade II shunt, and 15 had a grade I shunt. Six months after closure, 73 patients returned for c-TCD, and 14 (19.2%) had residual shunts: two patients had a grade IV shunt, three had a grade III shunt, and nine had a grade I shunt. Twelve months after closure, 58 patients returned for c-TCD, and 10 (17.2%) had residual shunts: one had a grade IV shunt, two had a grade III shunt, two had a grade II shunt, and five had a grade I shunt (Fig. 2). Of the 46 patients with residual shunts at 1 month after closure, 35 returned for a second visit during the 1-year follow-up period; by the end of study, residual shunts resolved in 24 patients, while six patients had a grade I shunt, two had a grade II shunt, two had a grade III shunt, and one had a grade IV shunt.

#### Residual RLS status and migraine relief

**Figure 3:**
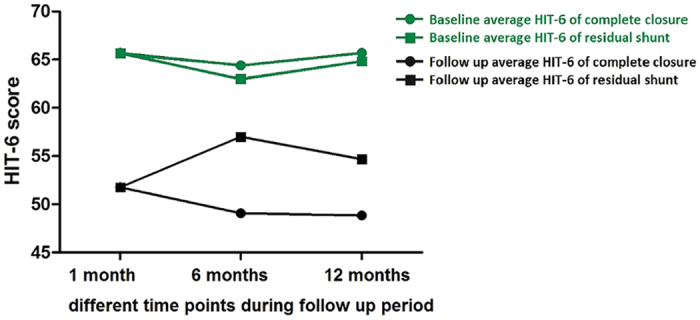
Comparison of migraine relief between patients with complete and incomplete patent foramen ovale closure at different time points during the follow-up period.

Among patients who returned for follow-up at 1, 6, and 12 months, we compared HIT-6 scores between those with and without residual shunts after PFO closure. At 1 month after closure, the average HIT-6 scores of 30 patients without residual shunts and 20 patients with residual shunts were 51.77 and 51.75, compared to average HIT-6 scores at baseline of 65.70 and 65.65, respectively. At 6 months after closure, the average HIT-6 scores of 30 patients without residual shunts and 8 patients with residual shunts were 49.07 and 57.00, compared to average HIT-6 scores at baseline of 64.40 and 63.00, respectively. At 12 months after closure, the average HIT-6 scores of 32 patients without residual shunts and 6 patients with residual shunts were 48.84 and 54.67, compared to average HIT-6 scores at baseline of 65.69 and 64.83, respectively (Fig. 3).

#### Evaluation of transcatheter PFO closure at the 12-month follow-up

**Figure 4:**
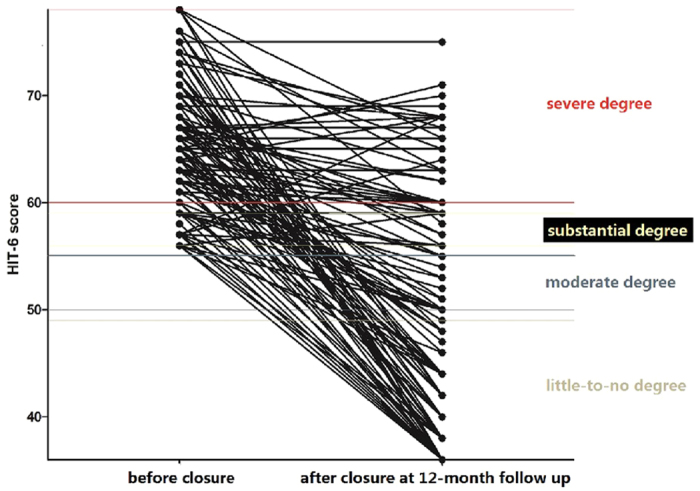
HIT-6 scores before and after transcatheter patent foramen ovale closure at 12-month follow-up, evaluated in the same patients.

HIT-6 scores before and 12 months after transcatheter PFO closure were evaluated in the same patients (Fig. 4). Sixty-seven patients (53.6%) who previously reported substantial- or severe-degree headache impact described little-to-no impact at the 12-month follow-up. Twenty-six patients (20.8%) reported HIT-6 score reductions to 36 (no headache); 16 patients (12.8%) reported reductions to a moderate degree of impact, and 42 patients (33.6%) still reported substantial or severe degrees of headache impact; of these, 26 patients reported no change from a severe degree of impact, two reported an increase to severe from substantial, five reported no change from a substantial degree of impact, and nine reported a decrease from severe to substantial. Thus, the degree of headache impact decreased in 92 patients (73.6%) decreased, remained unchanged in 31 patients (24.8%), and increased in two patients (1.6%).

### Comparison of long-term migraine relief between the transcatheter closure and control groups

#### Differences in post-procedure HIT-6 scores between groups

Mean post-procedure HIT-6 scores at the 12-month follow-up were 49.06 and 57.53 in the transcatheter closure group and control group, respectively. When these scores were adjusted for baseline HIT-6 scores, statistical significance was achieved between the two groups (48.77 vs. 57.85, p < 0.001). Covariates appearing in the model were evaluated at a baseline HIT-6 score of 64.31. The effect of the covariate (baseline HIT-6 score) on post-procedure HIT-6 score was also significant (p = 0.021).

Mean post-procedure HIT-6 scores at the 12-month follow-up for patients describing migraines were 51.29 and 57.44 in the transcatheter closure group and control group, respectively. When these scores were adjusted for baseline HIT-6 scores, statistical significance was achieved between the two groups (50.78 vs. 58.13, p < 0.05). Covariates appearing in the model were evaluated at a mean baseline HIT-6 score of 64.14. For patients describing migraines without aura, mean post-procedure HIT-6 scores at the 12-month follow-up were 48.23 and 57.55 in the transcatheter closure group and control group, respectively. When these scores were adjusted for baseline HIT-6 scores, statistical significance was achieved between the two groups (48.01 vs. 57.77, p < 0.001). Covariates appearing in the model were evaluated at a baseline HIT-6 score of 64.37.

#### Differences in change scores between groups

**Figure 5:**
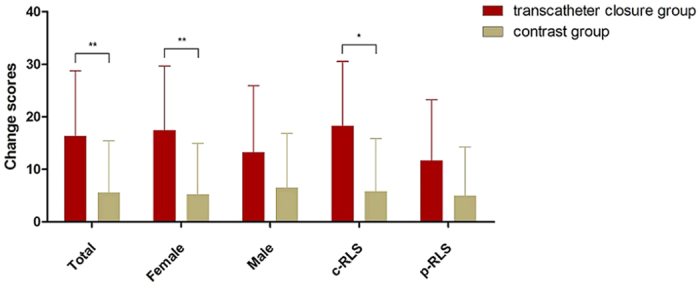
Differences in change scores between groups. Mean change scores were statistically different between the transcatheter closure group and the control group. In subgroup analyses, there were statistical differences in mean change scores between the transcatheter closure group and the control group except for the male and provoked RLS subgroups. *Indicates p < 0.05 and **Indicates p < 0.001.

The change score for each patient was calculated by subtracting the 12-month follow-up HIT-6 score from the baseline HIT-6 score. There was a statistically significant difference between the mean change scores of the transcatheter closure group and the control group (16.35 vs. 5.59, p < 0.001). HIT-6 scores were significantly decreased in the female transcatheter closure patient subgroup (17.46, p < 0.001) and the constant RLS subgroup (18.25, p < 0.05) (Fig. 5 and Table 2).

## Discussion

The goal of the EASTFORM study was to evaluate the effectiveness and safety of transcatheter PFO closure for the treatment of RLS-associated migraine in a Chinese population. The main findings of our study suggest that transcatheter closure might be a safe and effective approach for treating migraine in Chinese individuals, especially in female patients with constant RLS.

The relationship between PFO and migraine is controversial^21^. Indeed, data presented at cardiology conferences in 2006, 2014, and 2015 representing the MIST, PREMIUM, and PRIMA trials offered negative results regarding the utility of PFO closure for the treatment of migraine^22^,^23^,^24^. The present (EASTFORM) study evaluated the long-term impact of PFO closure on migraine in a real-world setting of patients enrolled and included in a continuous registry at a single centre in China, and importantly addressed ethnic differences in the efficacy and safety of PFO closure for migraine. Our study offers several advantages and disadvantages compared to previous randomized controlled trials. The use of a sham control procedure in the MIST and PREMIUM studies represents a correct methodological approach; however, the clinical situation in China did not permit the use of a sham control condition in our study. Strict inclusion criteria in the MIST and PRIMA studies allowed for the enrolment of migraine patients with aura and refractory status to medical treatment and the exclusion of patients suffering from severe chronic migraine. Alternatively, we enrolled patients suffering from substantial- or severe-degree migraine with moderate-to-large cardiac RLS, and grouped them according to the election or refusal of transcatheter PFO closure. The observation of changes in residual RLS status over a 12-month follow-up period in our study suggested that device endothelialisation was incomplete before 6 months, such that the 6-month follow-up period in the MIST study was likely insufficient. It is worth mentioning that the use of c-TCD in our study was important: c-TCD evaluation of residual shunts at the cerebral level is more directly relevant to migraine than TTE, as the probable mechanism of PFO-related migraine involves the arrival of agents into the cerebral circulation that are normally removed by the lungs. c-TCD has also been demonstrated to be a more reliable and sensitive screening method than TTE for RLS detection^25^. Therefore, the present study provides a novel and clinically relevant perspective on the use of c-TCD for the follow-up of PFO closure in migraineurs. Another important point is that the present study used the HIT-6 questionnaire to evaluate the migraine status. The HIT-6 score is a simple self-reporting evaluation system that covers six content categories (pain, social functioning, role functioning, vitality, cognitive functioning, and psychological distress) that are widely used in surveys of headache impact. The HIT-6 questionnaire has been previously validated as a useful tool for assessing headache-related disability in patients suffering from migraine^26^,^27^,^28^. We performed baseline self-evaluations of migraine impact prior to c-TCD screening to eliminate possible bias from the overestimation of pre-procedural migraine severity. Furthermore, to exclude the potential interferences of antiplatelet therapy, residual shunts (because of incomplete device endothelialisation), or migraine status measurement bias, patient HIT-6 scores were re-evaluated 1 year after baseline by the same neurologist (blinded to migraine status and group information) over the telephone.

Our study has important implications of using c-TCD in residual shunts evaluation. By comparing migraine relief in patients with complete versus incomplete closure at different time points during the follow-up period, we found similar mean HIT-6 scores at 1 month after closure and conjectured that placebo effects likely affected early judgments about headache relief. However, both the placebo effect and residual shunts were supposed to disappear later, as patients with incomplete closure had increased HIT-6 scores at 3 months and decreased scores at 6 months after closure. Of note, mean HIT-6 scores in patients with residual shunts were higher compared to those in patients without residual shunts throughout the follow-up period (except at 1 month after closure), which also suggested that residual shunts affected migraine relief. Taken together, these findings suggested that c-TCD is useful for evaluating the ability transcatheter PFO closure to treat migraine.

There were several limitations to the present study. First, it is difficult to overcome or account for the placebo effect without a sham control. We estimate the placebo effect to be approximately 35% in the present study, as the PREMIUM study showed that 32% of sham control patients with migraine responded positively with a 50% or more reduction in headache days. Patients in the transcatheter PFO closure group had the expectation of effectiveness, and a placebo effect was indicated by our data in the early follow-up period. Moreover, similar aspirin usage between the control group and the transcatheter closure group is not acceptable in a real world setting; thus, we compared long-term migraine relief between the two groups at 12 months, the quite long period which antiplatelet therapy and placebo effect did not appear to endure. Furthermore, the use of a patient-reported measure of migraine status is relatively subjective for an interventional study. However, migraine has several attributes that are subjective in nature and the HIT-6 scoring system has proven useful for the assessment of headache-related disability in previous studies^26^,^27^,^28^. One unexplained finding from our study was the report of new-onset severe visual aura (scintillating scotoma) with or without migraine after PFO closure in three patients. A previous study reported similar findings and speculated that nickel (Ni) in the PFO closure device may have precipitated this condition^29^. We took blood samples from two of these patients and found that the plasma Ni concentrations were 1.5 μg/L and 12 μg/L (normal reference standard: 2.98–7.34 μg/L). Without any treatment, visual auras decreased or resolved over time. Finally, our study did not investigate possible risk factors or predictors for residual shunts in patients after PFO closure. Future studies should investigate the possibility of undetected extracardiac RLS or special variable anatomies (such as long tunnel PFOs, “held open” PFOs, Eustachian valves, or Chiari networks) that might interfere with the deployment of occlusion devices^30^. Moreover, future device improvements have the potential to improve the safety and efficacy of the PFO closure procedure. Finally, there remain obstacles to the identification of patients with migraine who might benefit from PFO closure. To optimize the implementation of PFO closure in migraineurs, both anatomical and functional indicators of candidacy (e.g., dynamic cerebral autoregulation^31^ or biomarkers such as serotonin^18^) warrant investigation in future studies.

While migraine is generally non-life threatening, migraine combined with PFO can increase the likelihood of stroke. Currently, PFO closure is not a recommended procedure for the prevention or treatment of migraine headache, although some experts support a more proactive attitude towards PFO screening and closure in high-risk populations (e.g., patients with PFO-associated disorders such as migraine) and patients with high-risk hobbies/professions (e.g., weight-lifters, frequent flyers, and deep sea divers). A majority of clinicians remain conservative in their approach and require more definitive data on PFO closure before it can be considered as a plausible option for migraine treatment. Future studies will better inform the clinical relevance and benefits of PFO closure in migraine.

## Methods

### Participants

Between 2013 and 2015, 258 consecutive subjects were enrolled in the present non-randomized clinical trial. All subjects had a clinical history of migraine as diagnosed by a neurologist according to the International Classification of Headache Disorders III-beta^32^, and were refractory to or unwilling to use common migraine prevention medications. HIT-6 scores were assessed at baseline and were greater than 55 (substantial or severe degree of impact) for all included subjects. In addition, all included subjects exhibited c-TCD evidence of grade II−IV RLS and echocardiographic evidence of PFO. Subjects were excluded from the study if they had a history of seizure disorder or other organic central nervous system disease, headaches other than migraines (e.g., as a result of traumatic head or neck injury), or evidence of alcohol or substance abuse within the previous year. Furthermore, subjects were ineligible for transcatheter closure if they had a history of intracardiac thrombus or tumour, acute or recent (within 6 months) myocardial infarction or unstable angina, left ventricular aneurysm or akinesis, atrial fibrillation/atrial flutter (chronic or intermittent), another source of RLS identified at baseline (e.g., atrial septal defect and/or fenestrated septum), contraindication to aspirin or clopidogrel therapy, or if they were pregnant or had the desire to become pregnant within the next year.

**Figure 6:**
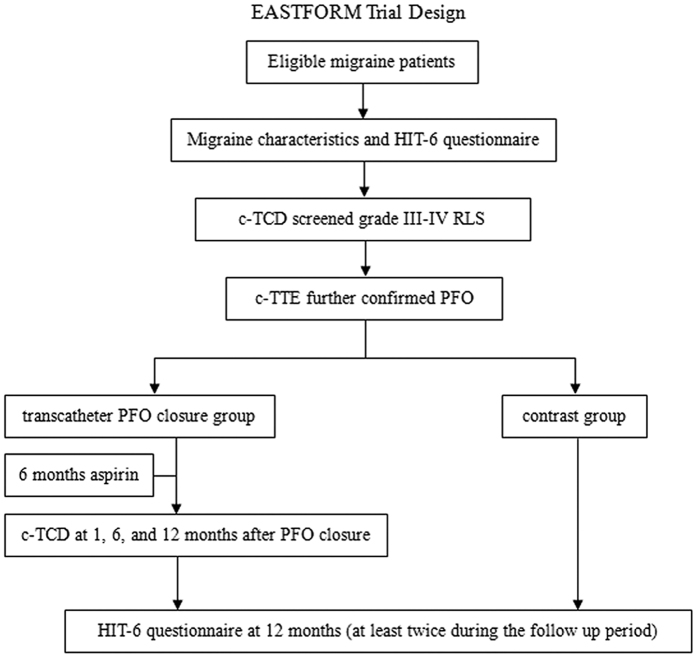
Schematic of the EASTFORM study protocol. Abbreviations: c-TCD, contrast-enhanced transcranial Doppler; c-TTE, contrast transthoracic echocardiogram; RLS, right-to-left shunt; HIT-6, Headache Impact Test-6.

In total, 130 patients who consented to transcatheter PFO closure were included in the transcatheter closure group, and 128 patients who refused the procedure were included in the control group. Migraine characteristics, questionnaires, and exam information were recorded using the Case Report Form at the ultrasound centre in the First Hospital of Jilin University. The study design (Fig. 6) was approved by the ethics committee of the First Hospital of Jilin University (clinical trial no. NCT02127294; registered on April 29, 2014; https://clinicaltrials.gov/ct2/show/NCT02127294). All patients provided written informed consent prior to participation. All methods were carried out in accordance with the approved guidelines.

### HIT-6

The HIT-6 questionnaire is a six-item self-report that was used in the present study to evaluate the severity of headache impact on patient activities of daily living, as previously described^26^,^28^. The degree of disability was categorized according to the obtained HIT-6 impact score as previously described^27^: little-to-no degree (HIT-6 score: 36–49), moderate degree (HIT-6 score: 50–55), substantial degree (HIT-6 score: 56–59), and severe degree (HIT-6 score: 60–78). We adopted a high minimally important change value of 6 or more points to establish a convincing relevant treatment effect^28^.

### c-TCD

c-TCD examinations were performed using a Multi-DopX4 TCD detector (DWL, Sipplingen, Germany) with monitoring of the left middle cerebral artery (MCA). An 18-gauge needle was inserted into the cubital vein with the patient in a supine position. The contrast agent was prepared by vigorously mixing 9 mL saline solution, 1 mL air, and a drop of the patient’s blood between two 10-mL syringes via a three-way stopcock^33^. After 30 mixing cycles, the contrast agent was rapidly injected as a bolus. Testing was performed once at rest and twice during the Valsalva manoeuvre (VM). A microbubble (MB) was defined as a visible and audible (click, chirp, or whistle), short-duration, high-intensity signal within the Doppler flow spectrum. RLS was defined as constant RLS if MBs were detected at rest and as provoked RLS if MBs were detected only after the VM. Based on the categorization of the International Consensus Criteria for RLS degree, a five-grade scale according to MB appearance in the TCD spectrum using unilateral MCA monitoring was adopted^34^: negative = no occurrence of MBs; grade I = 1–10 MBs; grade II = 11–25 MBs; grade III > 25 MBs, but no curtain; grade IV = curtain (single MBs were indistinguishable within the TCD spectrum).

### Transcatheter PFO closure

All procedures were performed by the same cardiac surgeon using a femoral approach under local anaesthesia in the operating room. A long sheath was advanced into the left atrium and a Cardi-O-Fix PFO device (STARWAY MEDICAL TECHNOLOGY, INC.) was used to occlude the PFO. The Cardi-O-Fix device is a self-expanding double-disk device made of nitinol wire mesh and polyester fabric. The device was sized according to the right atrial disk (18, 25, 30, or 35 mm), besides the same size of left and right atrial disk, the design also involved the left atrial disk smaller than the right atrial disk (18–25 mm; 25–35 mm) with a small central nitinol waist connector. The Cardi-O-Fix PFO device was placed and released via the long sheath under fluoroscopic guidance. Intracardiac echocardiography was also performed to evaluate PFO anatomy and correct device implantation. Heparin (80–100 U/kg) was administered during the procedure. Contrast transthoracic echocardiography (c-TTE), chest X-ray, and routine electrocardiography (ECG) were performed at 24 h after PFO closure, and aspirin (100 mg/day) was administered as antiplatelet therapy for 6 months following the procedure.

### Follow-up and study outcomes measures

The primary outcome measure was c-TCD follow-up (for the transcatheter closure group) within a 1-year period and the secondary outcome measure was HIT-6 scores for both groups to evaluate the long-term impact of PFO closure on migraine. Patients in the transcatheter closure group were required to perform c-TCD to assess residual shunts at 1, 6, and 12 months after the procedure. Device location and thrombus formation were reviewed and assessed on TTE. HIT-6 scores were assessed during the follow-up period using medical records or telephone interviews when records were not available. Each patient’s last follow-up HIT-6 score was evaluated 12 months after baseline by the same neurologist (who was blinded to migraine status and group information) through telephone interview. Patients were permitted to use rescue medication for the treatment of migraine attacks at any time.

### Statistical analysis

Statistical analysis was performed with SPSS 17.0 software (SPSS, Chicago, IL, USA). Categorical variables are reported as absolute values and continuous variables are summarized using descriptive statistics (mean ± standard deviation, SD) unless otherwise noted. HIT-6 scores, headache features, and residual shunts from medical records or telephone questionnaires were analysed. HIT-6 scores (before and after the procedure, between groups) were evaluated using an analysis of covariance, and change score differences between groups and subgroups were evaluated using independent t-tests after confirming that the data conformed to a normal distribution. Statistical significance was set at p < 0.05.

## Additional Information

**How to cite this article**: Xing, Y.-q. *et al*. Effectiveness and Safety of Transcatheter Patent Foramen Ovale Closure for Migraine (EASTFORM) Trial. *Sci. Rep.* **6**, 39081; doi: 10.1038/srep39081 (2016).

**Publisher's note:** Springer Nature remains neutral with regard to jurisdictional claims in published maps and institutional affiliations.

## Figures and Tables

**Table 1:** Demographic and clinical characteristics.

	Transcatheter closure group n = 125	Control group n = 116
Age, mean ± SD	39.0 ± 12.9	38.3 ± 12.2
Sex, F/M	92/33	83/33
Course, mean (range)	8.9 (1–40)	8.6 (1–40)
Migraine with aura, n	34	25
Visual aura	25	19
Sensory aura	6	4
Both	3	2
c-TCD, constant/provoked RLS	89/36	87/29
Atrial septal aneurysm, n	2	0
Baseline HIT-6, mean ± SD	65.4 ± 5.9	63.1 ± 5.8
Follow-up HIT-6, mean ± SD	49.1 ± 11.3	57.5 ± 9.6

c-TCD, contrast-enhanced transcranial Doppler; F, female; Headache Impact Test-6, HIT-6; M, male; RLS, right-to-left shunt; SD, standard deviation.

**Table 2:** Comparison of mean change scores between subgroups.

Mean change HIT-6 scores between baseline and 12-month follow-up			
	Transcatheter closure group	Control group	p-value
Females	17.46 (n = 92)	5.23 (n = 83)	p < 0.001
Males	13.27 (n = 33)	6.52 (n = 33)	p = 0.074
c-RLS	18.25 (n = 89)	5.80 (n = 87)	p = 0.002
p-RLS	11.67 (n = 36)	4.97 (n = 29)	p = 0.113

Abbreviations: c-RLS, constant right-to-left shunt; HIT-6, Headache Impact Test-6; p-RLS, provoked right-to-left shunt.
